# Time-Varying Insomnia Symptoms and Incidence of Cognitive Impairment and Dementia among Older US Adults

**DOI:** 10.1093/geroni/igab046.2470

**Published:** 2021-12-17

**Authors:** Nicholas Resciniti, Bezawit Kase, Valerie Yelverton, Matthew C. Lohman

**Affiliations:** 1 University of Southern California, Los Angeles, California, United States; 2 University of South Carolina, Columbia, South Carolina, United States

## Abstract

There is conflicting evidence regarding the association between insomnia and the onset of mild cognitive impairment (MCI) or dementia. This study aimed to evaluate if time-varying insomnia is associated with the development of MCI and dementia. Data from the Health and Retirement Study (n = 13,833) from 2002 to 2014 were used (59.4% female). The Brief Insomnia Questionnaire was used to identify insomnia symptoms compiled in an insomnia severity index, ranging from 0 to 4. In the analysis, participants’ symptoms could vary from wave-to-wave. Dementia was defined using results from the Health and Retirement Study (HRS) global cognitive assessment tool. Respondents were classified as either having dementia, MCI or being cognitively healthy. Cox proportional hazards models with time-dependent exposure using the counting process (start-stop time) were used for analysis. For each one-unit increase in the insomnia symptom index, there was a 5-percent greater hazard of MCI (HR = 1.05; 95% CI: 1.04–1.06) and dementia (HR = 1.05; 95% CI: 1.03–1.05), after fully adjusting. Using a nationally representative sample of adults aged 51 and older, this study found that time-varying insomnia symptoms are associated with the risk of MCI and dementia. This highlights the importance of identifying sleep disturbances and their change over time as potentially important risk factors for MCI and dementia.

